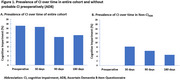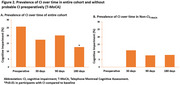# Prevalence and trajectory of postoperative cognitive impairment in older surgical patients: a multicenter longitudinal prospective cohort study

**DOI:** 10.1002/alz70857_098242

**Published:** 2025-12-24

**Authors:** Tony Tan, Ellene Yan, Leif Erik Lovblom, Jean Wong, Frances Chung

**Affiliations:** ^1^ Temerty Faculty of Medicine, University of Toronto, Toronto, ON, Canada; ^2^ Toronto Western Hospital, University Health Network, Toronto, ON, Canada; ^3^ University Health Network, Toronto, ON, Canada; ^4^ Dalla Lana School of Public Health, Toronto, ON, Canada; ^5^ Women's College Hospital, Toronto, ON, Canada

## Abstract

**Background:**

The COVID‐19 pandemic has accelerated the adoption of remote healthcare services, including cognitive screening tools delivered through virtual platforms. This study evaluates the prevalence and trajectory of cognitive impairment (CI) in older non‐cardiac surgical patients preoperatively and postoperatively at 30, 90, and 180 days, as assessed using remote cognitive assessment tools.

**Method:**

Following ethics approval, non‐cardiac surgical patients aged ≥65 years provided consent. Cognition was assessed virtually and over the phone using two validated screening tools preoperatively and at 30, 90, and 180 days postoperatively: the Ascertain Dementia Eight‐item Questionnaire (AD8) and Telephone Montreal Cognitive Assessment (T‐MoCA). Specifically, a cutoff score of ≥2 on the AD8 and ≤18 on the T‐MoCA suggested CI. Participants identified as having probable CI on these screening tools were subsequently referred to as having CI. A total of 244 patients completed the AD8 and 218 completed the T‐MoCA preoperatively and at least once postoperatively at 30, 90, or 180 days.

**Result:**

Among the 244 patients, the preoperative prevalence of CI on the AD8 was 17%, which did not change significantly after surgery (Figure 1A). Of the 202 participants without preoperative CI on the AD8, the incidence of CI was 8.3%, 6.4%, and 4.6% at 30, 90, and 180 days postoperatively, respectively (Figure 1B). In contrast to the AD8, the T‐MoCA revealed a 26% prevalence of CI preoperatively, which significant decreased at 180 days (13% vs. 26%, *p* = 0.01; Figure 2A). Among participants without preoperative CI on the T‐MoCA, a similar trend to AD8 was observed, with the postoperative incidence rates of CI being 11.1%, 7.8%, and 8.0% at 30, 90, and 180 days, respectively (Figure 2B).

**Conclusion:**

Regardless of the cognitive assessment tool, we found that participants without CI preoperatively experienced an increase in CI at 30 days postoperatively, followed by a decline at 90 and 180 days. Approximately 5% of patients developed CI on the AD8, and 8% on the T‐MoCA at 6 months postoperatively. These findings highlight the potential impact of surgery on older adults with normal cognitive function and emphasize the value of remote cognitive screening tools in monitoring postoperative cognitive changes and identifying at‐risk patients.